# High-fidelity national carbon mapping for resource management and REDD+

**DOI:** 10.1186/1750-0680-8-7

**Published:** 2013-07-16

**Authors:** Gregory P Asner, Joseph Mascaro, Christopher Anderson, David E Knapp, Roberta E Martin, Ty Kennedy-Bowdoin, Michiel van Breugel, Stuart Davies, Jefferson S Hall, Helene C Muller-Landau, Catherine Potvin, Wayne Sousa, Joseph Wright, Eldridge Bermingham

**Affiliations:** 1 Department of Global Ecology, Carnegie Institution for Science, 260 Panama Street, Stanford, CA 94305, USA; 2 Smithsonian Tropical Research Institute, Apartado 72, Balboa, Republic of Panamá; 3 Department of Biology, McGill University, 1205 Docteur Penfield Ave, Montreal, Canada; 4 Department of Integrative Biology, University of California, 1005 VLSB, Berkeley, CA 94720-3140, USA

**Keywords:** Biomass, Carbon stock, Carnegie Airborne Observatory, Deforestation, Forest degradation, Forest inventory, Light Detection and Ranging, Panama

## Abstract

**Background:**

High fidelity carbon mapping has the potential to greatly advance national resource management and to encourage international action toward climate change mitigation. However, carbon inventories based on field plots alone cannot capture the heterogeneity of carbon stocks, and thus remote sensing-assisted approaches are critically important to carbon mapping at regional to global scales. We advanced a high-resolution, national-scale carbon mapping approach applied to the Republic of Panama – one of the first UN REDD + partner countries.

**Results:**

Integrating measurements of vegetation structure collected by airborne Light Detection and Ranging (LiDAR) with field inventory plots, we report LiDAR-estimated aboveground carbon stock errors of ~10% on any 1-ha land parcel across a wide range of ecological conditions. Critically, this shows that LiDAR provides a highly reliable replacement for inventory plots in areas lacking field data, both in humid tropical forests and among drier tropical vegetation types. We then scale up a systematically aligned LiDAR sampling of Panama using satellite data on topography, rainfall, and vegetation cover to model carbon stocks at 1-ha resolution with estimated average pixel-level uncertainty of 20.5 Mg C ha^-1^ nationwide.

**Conclusions:**

The national carbon map revealed strong abiotic and human controls over Panamanian carbon stocks, and the new level of detail with estimated uncertainties for every individual hectare in the country sets Panama at the forefront in high-resolution ecosystem management. With this repeatable approach, carbon resource decision-making can be made on a geospatially explicit basis, enhancing human welfare and environmental protection.

## Introduction

Carbon accounting has reached the vanguard of national resource management. The carbon stored in vegetation and soils is a vitally important component of national greenhouse gas mitigation strategies [1], and abrupt changes in carbon storage can indicate interruptions of other ecosystem services such as water quality and biodiversity [2,3]. Despite the widely recognized importance of carbon storage in ecosystems, geospatially explicit mapping and monitoring of carbon stocks has remained a challenge, largely due to the natural heterogeneity of vegetation structure, diffuse and ubiquitous patterns of land-use change, and inexact techniques and technologies for carbon measurement [4,5]. Geospatially explicit carbon accounting would provide enormous benefits for national resource monitoring, and would greatly accelerate international agreements on carbon emissions, such as REDD + (Reduced Emissions from Deforestation and Forest Degradation), which must be implemented with confidence among participating countries [6,7].

Traditionally, national-scale carbon monitoring has been accomplished with networks of field inventory plots [8]. Much effort and expense has been applied to install and monitor such plots, yet they often prove difficult to maintain over time. Furthermore, plot networks offer direct measurement of a tiny amount of actual forest [9], without an ability to report on spatially explicit carbon stocks and changes in those stocks (emissions). In response to this challenge, there has been rapidly growing interest in the use of geospatial mapping technologies to augment field plot inventories [10], and several new approaches have emerged to extend plot-based carbon estimates to millions of hectares [11], and even globally [12,13]. Airborne laser technology called Light Detection and Ranging (LiDAR) stands apart in this effort because, like field inventories, LiDAR measures aspects of the physical structure of woody vegetation in ecosystems ranging from sparse shrublands to dense forests [14-17].

Even with the advent of airborne LiDAR as a game-changing tool for estimation and monitoring of aboveground carbon stocks, reducing cost in its application at national scales requires approaches that integrate LiDAR sampling measurements of vegetation structure with full-coverage satellite data. Testing has demonstrated that this is best done using spaceborne optical and radar sensors imaging at 10-m to 1-ha spatial resolution in order to resolve fine-scale variation in vegetation cover and condition [18,19]. These higher resolutions, while becoming increasingly routine at the national level [19], remain mostly for the future in operational global-scale monitoring [20]. With the upscaling step from LiDAR to full-coverage satellite maps, however, uncertainty is introduced on the per-hectare basis, and this uncertainty has remained extremely difficult to assess in a geospatially explicit manner. As a result, questions persist over the per-hectare reliabilities of national-scale carbon stock monitoring [21], and this issue continues to stand in the way of what could be a strong economic forcing mechanism to mitigate climate change.

**Figure 1:**
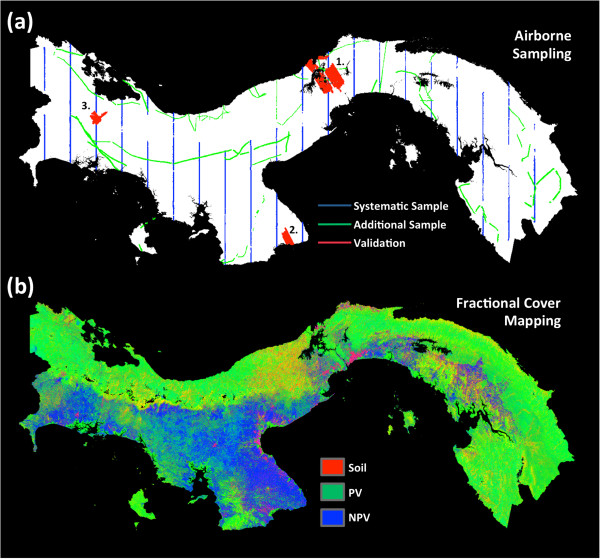
**Major remote sensing inputs to the national carbon mapping study.** (**a**) LiDAR flight coverage obtained by Carnegie Airborne Observatory, including systematic sampling designed to achieve robust coverage of national ecosystems, plus additional samples added as time and weather permitted, and three large validation areas set aside to evaluate national mapping approaches. (**b**) CLASlite fractional cover based on a mosaic of 158 Landsat scenes, and MODIS data in areas of poor Landsat coverage. Image is a red-green-blue composite, with % bare soil or substrate coverage shown in red, % photosynthetic vegetation (PV) shown in green, and % non-photosynthetic vegetation (NPV) shown in blue.

Using a new combination of techniques, we developed a high-resolution nationwide map of aboveground tree carbon stocks, referred to as aboveground carbon density (ACD), with geospatially explicit uncertainty estimates for the Republic of Panama. This paper focused exclusively on ACD of standing trees ≥ 10 cm in diameter, and not on belowground carbon, necromass, or lianas and small woody plants. Although Panama is a relatively small nation of 7,551,700 ha, the country contains a vast array of environmental conditions and a complex mosaic of land-use histories, resulting in a wide range of tropical vegetation types and carbon stocks (Figure 1). Our goal was to integrate and test the accuracy of methods for scaling ACD estimates from field plots, to airborne LiDAR measurements, to the national level using high-resolution satellite imagery. Critically, we estimate and map the uncertainty of carbon stocks in every hectare of Panama. The hectare has proven to be a reliable unit for carbon stock error estimation [22], and it is the most common unit of land utilization and ecological condition in science, conservation, management and resource policy development activities [5,19,23].

## Results and discussion

### Airborne LiDAR mapping

**Figure 2:**
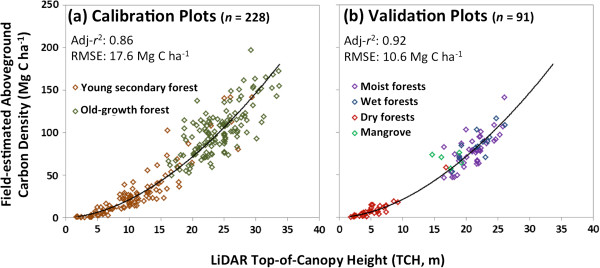
**Linking airborne LiDAR to field-based estimates of aboveground carbon stocks.** (**a**) Calibration of LiDAR top-of-canopy (TCH) height to field-plot estimates of aboveground carbon density [ACD = 0.359*TCH*^1.7676^]. (**b**) Separate validation plots in four ecoregions were compared against the calibration model, which is the black line in both panels. For the validation plots, RMSE (predicted versus observed) represents an estimate of LiDAR-calibration uncertainty, given that the field plots are not randomly distributed across the study area.

Top-of-canopy height (TCH) was measured at 1.1 m spatial resolution in systematically collected, national LiDAR sampling transects covering a total of 391,857 ha throughout Panama (Figure 1). Calibration of airborne LiDAR TCH measurements to estimated aboveground carbon density (ACD) in 228 field plots ranging in size from 0.1-0.36 ha are highly predictive of field-estimated ACD (adj-r^2^ = 0.86, RMSE = 17.6 Mg C ha^-1^) across a range of vegetation types, from forests to grasslands, and across wide-ranging environmental conditions (Figure 2a, Additional file 1: Table S1; Additional file 2: Figure S1). Furthermore, 91 additional plots (0.1-1.0 ha) set aside and used solely for validation are even more tightly related to LiDAR-estimated ACD (adj-r^2^ = 0.92, RMSE = 10.6 Mg C ha^-1^) (Figure 2b, Additional file 2: Figure S2). These LiDAR-to-ACD estimates fall well within the error range of the plot-based estimates, and critically they approach 10% error at 1-ha plot scale.

### National mapping

**Figure 3:**
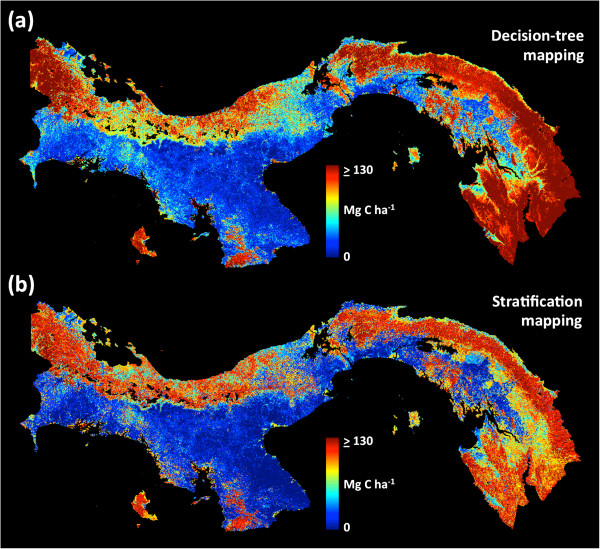
**National**-**scale mapping of aboveground carbon density** (**ACD**) **using two techniques.** (**a**) Decision-tree based mapping with the RandomForest algorithm; (**b**) Stratification of satellite input variables into 2039 unique classes.

**Figure 4:**
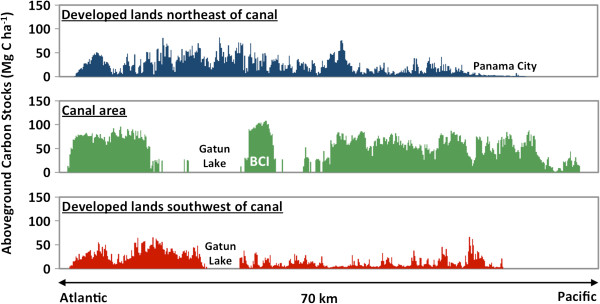
**The mark of more than a century of land development in Panama, contrasted with forest conservation within the area of the canal.** The canal area (center panel) contains some old-growth forests such as on a portion of Barro Colorado Island (BCI), with carbon stocks > 100 Mg C ha^-1^, and extensive areas of mature secondary forests with carbon stocks nearly as high. To the northeast and southwest of the canal area, carbon stocks are greatly suppressed due to land-use change, particularly on the Pacific side near Panama City.

Decision-tree and stratification approaches yielded similar national-scale maps of aboveground carbon densities (Figure 3). Analyses conducted on LiDAR-scale ACD maps and satellite data quantitatively link elevation, slope, climate and fractional canopy cover to carbon storage patterns (Additional file 2: Figures S3-S6). The highest ACD levels are found in humid forests on the Caribbean side of the continental divide running east–west throughout the country. These forests cover approximately 2,000 km^2^ of Panama, and where they are undisturbed, their ACD levels often exceed 100 Mg C ha^-1^. Moreover, large tracts of forest to the east in the Darien region near Colombia, as well as in the Panama Canal area (Figure 4), and a few smaller areas on the Pacific side, also contain substantial carbon stocks. With few exceptions, the large remaining tracts of contiguous forest in Panama are contained within areas set aside for conservation, canal watershed protection, or within indigenous territories.

In contrast to the high biomass forests, large regions of the country are comprised of deforested land with very low carbon stocks. These areas are primarily found to south of the continental divide on the Pacific side of the country, and in large developed corridors outside of the protected watershed of the Panama Canal (Figure 4). Additionally, a relatively narrow stretch of Caribbean coastal community harbors suppressed carbon stocks, particularly in the west near Bocas del Toro and in Guna Yala to the east. Finally, there is a region to the west of the Panama Canal that, while still forested, has been degraded by development, displaying ACD levels that are lower than neighboring forests in similar physiographies (Figure 3).

### National map validation

**Figure 5:**
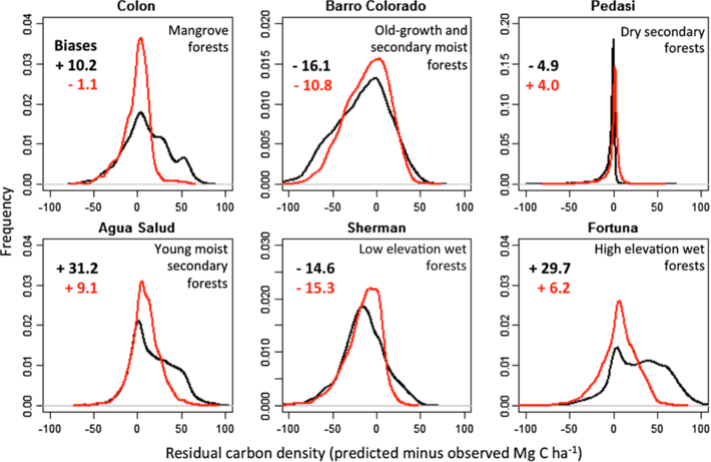
**LiDAR**-**based validation for six distinct ecoregions in Panama.** For each region, more than 1,000 ha of LiDAR-estimated ACD data are compared to the stratification (black) and decision-tree (red) modeling results for national-scale ACD maps. Biases (national minus LiDAR-scale) are ≤ 15.3 Mg C ha^-1^ for all regions using the decision-tree based approach.

Using six separate ecoregions, each at least 1,000 ha in size (see Methods), we quantitatively compared distributions of vegetation ACD derived with LiDAR against those extrapolated to the national scale with satellite data and modeling (Figure 5, Additional file 2: Figures S7 and S8). The decision-tree approach for national upscaling provides a better result, with mean biases (net difference in national mapping and LiDAR-scale carbon) ≤ 15.3 Mg C ha^-1^ in all six ecoregions (Figure 5). Despite low biases, some regions are noisier where satellite coverage is poor due to persistent cloud cover (e.g., Additional file 2: Figure S8). In all but one case, biases are larger when using the upscaling approach based on stratification, particularly in high elevation wet forests (Figure 5). Given these findings, we carried forward only the decision-tree based approach for the remaining analyses.

**Figure 6:**
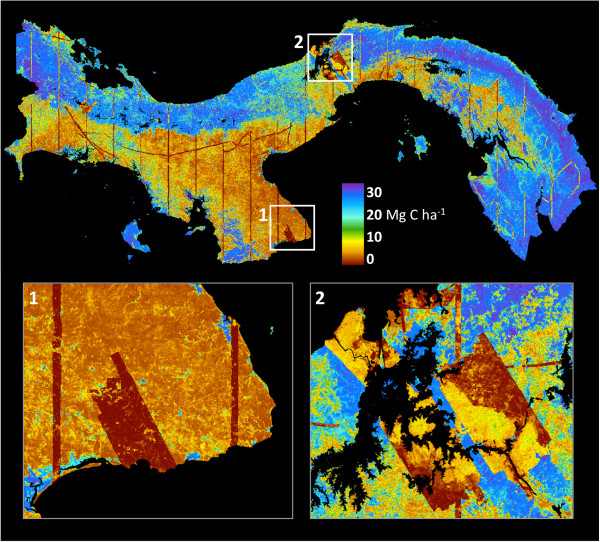
**Geospatially explicit uncertainty estimates at 1-ha resolution based on nested field**-**plot**, **airborne LiDAR**, **and decision**-**tree modeling methods.** Note that direct LiDAR-observed areas (e.g., thin transects, and large polygons in insets 1 and 2) achieve extremely low uncertainties compared to areas upscaled to the national level using models.

To estimate per-hectare uncertainties, we modeled the pixel-level relationship between errors in LiDAR validation regions versus the national-scale ACD estimates (see Methods). We propagated these estimated mapping errors using field plot-to-LiDAR calibration errors that we established during validation (10% at 1 ha resolution; Figure 2b), using the square root of the sum of squared errors in units of carbon per hectare. Due to heteroskedasticity common to carbon stock errors at both the tree and plot scales [24-27], the resulting mapping errors follow the same pattern, with larger errors in regions of greater mapped carbon density (Figure 6, Additional file 2: Figure S9). In the national map, these range from << 5 Mg C ha^-1^ in lower-biomass regions to > 30 Mg C ha^-1^ in regions harboring the highest carbon stocks. Critically, however, LiDAR-scale estimates of carbon stock, which alone cover 4% of Panama (see lines in Figure 6), have far lower map-scale uncertainties of about 2 Mg C ha^-1^ in the lowest biomass regions and < 15 Mg C ha^-1^ in most high-biomass forests (> 100 Mg C ha^-1^) (Figure 6).

Our results demonstrate the accuracy, relative to exhaustively measured field plots, of using airborne LiDAR for estimating aboveground carbon densities across vegetation types ranging from grasslands to dense tropical forest in Panama. The LiDAR-based carbon mapping uncertainty of about 10% at 1-ha resolution has now been demonstrated empirically in several tropical forest LiDAR studies [28,29] as well as in a recent metaanalysis [17], but rarely has a large number of field plots been available for completely independent validation of the LiDAR calibration, as we have shown here. Critically, this 10% mismatch between LIDAR-based and plot-based estimates of ACD at 1-ha resolution is within the range of the uncertainty reported for field plots alone, which can reach 20-30% [30,31].

At this point, the challenge in decreasing uncertainty rests with improving the field techniques, which will require more emphasis on measuring real plot-level forest biomass instead of estimating biomass from traditional field inventories with allometric equations [4,32]. More work should also go into developing improved conversions between biomass and carbon [33]. As efforts expand to improve plot-based ACD estimates, their calibration against LiDAR data will also improve and will further reduce the overall uncertainty of LiDAR-assisted carbon mapping. Importantly, although improvements in the field-based stem allometries underlying LiDAR calibrations would alter the overall levels of geospatially-estimated ACD, such changes would affect both field-based and LiDAR-based predictions in concert, with little to no influence on relative spatial uncertainties [4,34]. In general, our results show that LiDAR approaches can stand in for field plots, both in humid tropical forests and among drier tropical vegetation types.

The value of geospatially explicit carbon mapping is further expressed at the national level. As the LiDAR approaches replace field inventories as a primary estimator of aboveground carbon stocks, the enormous regional sampling provided by airborne LiDAR surveys allows for improvements in linking mapped environmental variables and land-use data to mapped carbon stocks. From this step, the underlying environment-to-ACD relationships that then support stratification, decision-tree analysis or other methods of upscaling become more robust, and as result, the national maps become increasingly reliable. Our analyses indicate that the fractional cover of photosynthetic and non-photosynthetic vegetation from Landsat imagery, along with topography and climate data, play a contributing role in mapping national-scale ACD with low uncertainties on a per-hectare basis (Additional file 2: Figure S4, S6). With the decision-tree approach, for example, our estimated uncertainties average 20.5 Mg C ha^-1^ at the national level. In deforested and dryland regions harboring very low carbon densities (< 20 Mg C ha^-1^), uncertainties fall to a level commensurate with that of small shrubs and grass cover (≤ 5 Mg C ha^-1^) [35].

As a whole, we demonstrate a powerful analytical chain, from well-measured field plots, to high-resolution LiDAR, and to satellite and environmental data, that achieves national-scale carbon stock maps with high fidelity and low per-hectare uncertainty. This has not been possible using field or global satellite approaches alone. Whereas field-based approaches cannot resolve the spatial distribution of carbon stocks, global satellite approaches do so with lower spatial resolution – currently 25 to 100 ha per mapping cell [12,13]. To date, the global benchmark maps do not provide per hectare or per-pixel uncertainty, and where they have been compared, the approaches diverge by up to 100% on any given 25–100 ha land parcel [36]. Nonetheless, the global approaches do converge at biome, country and globally-integrated levels [37], making them valuable for countries still working toward high-resolution carbon mapping with geospatially-explicit uncertainty reporting.

We note that our error maps should be viewed as estimates. Actual carbon mapping errors can only be truly assessed with totally independent, direct carbon mass measurements, which themselves can only be accomplished with plot-scale destructive harvests. Until such time, our estimated errors are reasonable in that they are derived through a combination of field-plot and LiDAR-scale validation. In each case, the data used to estimate errors were completely excluded from the project until the validation phase.

The spatial detail provided by our approach opens new doors to understanding environmental and human controls over the Earth’s aboveground carbon stocks. Topography, climate and geologic substrate impart an ecologically nested set of patterns in ACD [38-41]. That is, the natural background patterns are far from homogeneous, and in fact, display geographical variances at multiple, nested scales. In Panama, this is clearly expressed with elevation on a regional basis, as well as with slope on a local or sub-watershed basis. For example, aboveground carbon stocks are highest in forested areas to the north of the continental divide, with these regions containing localized carbon-storage hotspots on moderate slope angles [40]. Superimposed on these non-random, ecologically nested patterns is the expansive footprint of human activity – the overwhelming driver of ACD patterns in Panama. At the national level, it is clear that vast portions of the country, particularly in the south, have been deforested or degraded. Yet on a landscape scale, the pattern is more complex, expressing variation in land-use decisions that co-occur with catenas and other fine-scale hillslope patterns. These findings become obvious due to the high-resolution mapping achieved.

The high fidelity of the final carbon map sets Panama apart from every other country today. At both jurisdictional and international levels, environmental policy and management can utilize high-resolution carbon maps to undertake activities on a geospatially explicit basis. As a result, monitoring and verification reach a new level of competency, affording more comprehensive actions to enhance human welfare and environmental protection. Although United Nations efforts to reduce emissions from deforestation and forest degradation have been slow to develop in the international arena, jurisdictional-scale REDD + activities (as well as voluntary projects) require high-fidelity carbon maps to raise the per-hectare value of ecosystem carbon stocks and avoided emissions. With a high-resolution carbon basemap such as we have developed for Panama, the technical and financial hurdles to monitoring emissions shrink to repeat satellite mapping from largely free data and automated methods [11]. Doing so allows for monitoring of carbon losses from and gains to the carbon basemap using medium to high resolution optical satellite data such as from the Landsat series going back to 1982 and continuing today [29]. This approach was greatly enhanced with the successful deployment of Landsat 8 on February 11, 2013; all Landsat data are free to the global community. Moreover, the European Sentinel satellite series will eventually provide similar data streams.

Increasing satellite data availability provides strong leverage to transfer and scale our approach to other nations and jurisdictions. The cost of satellite data acquisition, processing and analysis has recently plummeted, based mostly upon free data sources and analytical methods e.g., [42,43], to a level requiring perhaps two to five trained technicians, depending upon the size and environmental complexity (e.g., topography, land use) of the tropical country. A good example is the Peruvian government, which went from little government-led deforestation monitoring in 2008 to transparent monitoring by 2012 with a small group of geospatially-trained technicians [44]. Additionally, field plots are extremely expensive to establish and maintain, for example, costing the Carnegie Institution (a non-profit with no program overhead costs) from $2000-$5000 USD per hectare in basic plot setup and measurement. Minimizing the use of plots is thus a serious cost consideration, but it must be done tactically from both an ecological-sampling and a LiDAR calibration-validation standpoint. In response to this, Asner et al. [15] developed more general equations to convert LiDAR measurements to estimates of ACD for a very wide range of tropical vegetation types and land-use conditions. Although this approach has been demonstrably successful in remote regions such as the Colombian Amazon [38], validation plots remain highly valuable for increasing accuracy and transparency. Finally, the airborne LiDAR component is both scalable and cost effective, if deployed appropriately. First, there are a multitude of airborne LiDAR providers spread around the world (e.g., http://www.airbornelasermapping.com/ALMID.html). It thus becomes the responsibility of the carbon-mapping technicians (often the same people doing the satellite monitoring) to direct the LiDAR data collection according to the more robust and efficient approach available based on geostatistical and logistical constraints. For Panama, we selected a systematic aligned sampling scheme that was highly efficient by crossing the major ecoregions and land-use conditions arrayed throughout Panama. Our sampling also precisely overlapped with the long-term plot network plans of the U.N. Food and Agriculture Organization (FAO). Had we only focused on LiDAR data acquisition for this project, our costs for data collection and analysis would have been less than $600,000 USD, or about $1.00 USD per hectare covering nearly 600,000 hectares for national “inventory”, plus calibration and validation. With airborne LiDAR, there is also an economy-of-scale effect, whereby larger projects become much less expensive on a per-area basis. In the Colombian Amazon [38] example covering a region more than twice the size of Panama, the cost for airborne LiDAR acquisition and analysis was about $0.15 USD per hectare. These, and yet other financial and logistical issues, will affect the transferability and scalability of our high-resolution mapping approaches throughout the world.

Beyond REDD + and other carbon-policy mechanisms, the utility of high-resolution aboveground carbon maps within countries remains largely underappreciated at present. First, vegetation carbon stock is a surrogate for many other ecosystem services. For example, high-biomass forests enhance water quality, provide erosion control, and stabilize water flow through ecohydrological mechanisms [45]. In other landscapes, including agricultural lands, the often-complex spatial distributions of woody carbon stocks serve as a habitat quality indicator for higher trophic species [46]. The geography of carbon storage is one of the clearest ecological metrics of habitat suitability in fragmented tropical forest landscapes. Other potential applications for high-fidelity carbon mapping at the national scale will continue to be identified as the analytical approaches reported here further improve and flourish in the years ahead.

## Methods

### Study region

The Republic of Panama provides a unique opportunity to develop and test geospatially explicit approaches for estimating aboveground carbon density (ACD) at multiple geographic scales. A combination of geology, topography, regional climate and a storied land-use history have resulted in a complex mosaic of vegetation types ranging from open shrublands to dense tropical forests [47,48]. Steep spatial gradients of vegetation type and cover exist throughout the country, but particularly across the continental divide, from the wet northern Caribbean coast to montane cloud forests to drier and highly deforested Pacific drylands (Figure 1).

### Overall design

We developed national spatially explicit maps with uncertainties of ACD at 1-ha resolution. To do so, the project was carried out in three major activities: (i) field plot measurement and ACD estimation for LiDAR calibration and validation; (ii) airborne LiDAR data acquisition and processing; and (iii) national upscaling from the LiDAR coverage using two comparative modeling approaches with the same satellite data. Airborne LiDAR sampling was carried out in January-February 2012 using an approach based on systematically aligned transects of 1.5 km swath width (Figure 1a). These transects were selected to match a planned national forest inventory plot network, to be installed in the coming years by the United Nations Food and Agricultural Organization (FAO). Extra LiDAR coverage was acquired in areas prone to cloud cover and whenever weather and time permitted. The LiDAR data were converted to vegetation top-of-canopy height (TCH) at 1-ha resolution, and then modeled against field plot-aggregate estimates of ACD. A large network of field plots was used for calibrating or validating relationships between plot-scale ACD and LiDAR TCH (Additional file 2: Figures S1 and S2). The derived LiDAR-scale ACD maps were then integrated with satellite-based measurements of vegetation cover and condition, topography and precipitation to model carbon stocks at 1-ha resolution at the national level (Figure 1b, Additional file 2: Figure S3). The integration of LiDAR-scale ACD and satellite data was accomplished using two modeling approaches – a decision tree algorithm and regional stratification.

### Plot-based carbon stocks

We integrated field plot data from five distinct ecotypes in Panama (Additional file 2: Figure S1, Additional file 1: Table S1): We calibrated our LiDAR data with field-based carbon estimates from (1) old-growth moist forests on Barro Colorado Island [BCI; 40] and (2) secondary moist forests within Agua Salud [AS; 40, J. Hall and M. van Breugel, unpublished data, 49], and subsequently compared the predictions of this model to additional field-based estimates of carbon in (3) mixed old-growth and old secondary wet forests at Fort Sherman (SHRM), (4) dry secondary forests on the Azuero Peninsula (AZ; J. Hall and M. van Breugel, unpublished data) and (5) mangrove forests in Colon (COL) [49].

Carbon stock estimation from field data was made using a consistent methodology across all plots; an accounting of allometric models is provided in Additional file 1: Table S2. We limited measurements to standing woody stems ≥ 10 cm in diameter. For allometric estimation of aboveground biomass, we applied the general framework of Chave et al. [25] “model 1”, which includes input variables for height and wood density, and we used 48% of dry biomass as the conversion to carbon units [33]. Although a localized secondary forest allometry is available for AS [50], the model lacks a height input variable. While simple models are more parsimonious from a statistical point of view (i.e., when fitting a model), the models subsume height variation into the coefficients, making them more prone to bias in another region if the relationship between diameter and height varies from the built-in assumption of the coefficients. Recent analysis of the behavior of allometric models in the tropics suggests that height is essential as an input variable; without it, large over-estimations are likely [51]. We used the Chave et al. approach combined with a locally constructed diameter-to-height model for all plots in BCI, AS, and SHRM. In the case of AS, we found that the results of the Chave et al. model 1 approach were consistent to those of Breugel et al. [50]. Note that Breugel et al. [50] previously found that Chave et al. model 2 – which also lacks a height input variable – overestimated carbon stocks in AS.

Inventory-based height information was not available for either the AZ or COL validation plots, and thus we utilized the LiDAR data to constrain the maximum possible diameter-to-height relationship and minimize overestimation [sensu 51]. Specifically, we fit the maximum height within the LiDAR data for each plot to the maximum measured diameter for the same plot in the field to obtain an estimated height-diameter allometry for inclusion in the biomass equations (Additional file 2: Figure S2). We viewed this as a conservative method to constrain our field-estimated carbon stocks for tropical dry forests and mangroves in Panama. This step was essential to prevent unrealistically high height estimations for dry and mangrove trees that would result from the application of our existing Panama height to diameter relationships [51].

For wood density, we used local information first (e.g., species-specific wood density samples taken in the field), followed by increasingly general approaches with decreasing taxonomic resolution as needed from the Chave et al. [52] database. Ultimately, absent a wood density value for a species or genera, a default regional wood density value of 0.56 for Central America was used [52].

We note that allometry-based ACD estimation, whether done in the field or from LiDAR, is not the same thing as measuring carbon mass [4]. Yet, allometry is one of the most conserved properties in nature [26], and until whole-plot harvests enable direct measurement of carbon stock, allometry will continue to play the major role in carbon stock estimation and mapping. We also note that any departures from allometric-estimated carbon stocks in real forests will affect both field- and LiDAR-based carbon estimation in parallel [34].

### LiDAR data

For calibration, the LiDAR data were collected using the Carnegie Airborne Observatory (CAO) Alpha [53] or AToMS [54] sensor packages in 2009 and 2012, respectively, with data collection and analysis methods applied consistently (Figure 1). Both the Alpha and AToMS LiDAR sensors are full waveform, but the work presented here relied only on the discrete return data of up to four returns per pulse in order to make the results applicable to a much wider range of LiDARs currently in operation throughout the world. Both CAO LiDARs were operated at 2,000 m above ground level with 1.1 m spot spacing, a 30^o^ field of view, and a pulse repetition frequency of 50 kHz, for which the aircraft maintained a ground speed of < 110 knots. Both LiDARs have a laser beam divergence of 0.56 mrad (1/e). Despite the consistency of data collection parameters, the Alpha and AToMS LiDARs differed in laser diode power and laser receiver sensitivity. For this reason, the AToMS LiDAR proved much more sensitive to the internal 3-D architecture of any given forest canopy.

Although Asner et al. [15] previously compared regions using LiDAR-derived mean canopy profile height (MCH), we used top-of-canopy height (TCH) in this study. We did so for two reasons: (1) while TCH is available for all airborne and spaceborne LiDARs, MCH is not available from many systems, and (2) MCH is much less generalized across sensor types than TCH, owing to canopy penetration differences [16]. TCH was determined by constructing ground and surface digital elevation models [55], and subtracting them to determine height at 1.1 m resolution. The average of all 1.1 m pixels for which the center of each pixel was contained within a plot footprint was used as a measure of TCH for each field plot.

We used maximum likelihood analysis to fit a power-law model between field-estimated carbon stocks and LiDAR-measured forest height. The fit was performed on the un-transformed TCH and ACD values using a non-arithmetic error term to account for heteroskedasticity; this method is analogous to fitting a linear model to the log-transformed TCH and ACD data, but avoids the need for back-transformation [27]. The model was fit using young secondary forest plots from Agua Salud and mature moist forest plots from Barro Colorado Island and LiDAR data from 2009: ACD = 0.359 × TCH^1.7676^, resulting in an adjusted R^2^ = 0.86 and RMSE = 17.6 Mg C ha^-1^. The model was subsequently validated with 91 additional plots from dry forests in Azuero, older secondary forests in Gigante, mature wet forests at Sherman, and mangrove forests in Colon together with LiDAR data from 2012, yielding an adjusted R^2^ = 0.92 and a RMSE (predicted versus observed) = 10.6 Mg C ha^-1^ (plot details in Additional file 1: Table S1). That the validation model would perform better than the calibration model is not surprising; plot sizes were generally higher in the validation dataset, and previous work has shown that errors approach 10% at 1-ha plot size [17,22].

Mangroves showed a small under-prediction by the model (Figure 2b); while the limited number of plots precluded a diagnosis at this time, higher wood density is partly responsible. The results are consistent with Asner et al. [15], which found that variation in wood density and forest stocking (basal area) for the same LiDAR-measured height was primarily floristic and biogeographic in origin, producing modest disagreement only at very broad scales (e.g., Hawaii versus the Neotropics), rather than within a region.

### Satellite data

We produced a dry-season composite using 158 Landsat 5 and Landsat 7 (SLC-off) images from the months of December through March, for the years 2008–2012 (Figure 1b). After all images were separately processed by CLASlite [42] to mask clouds and apply radiometric correction, we applied a pixel-mosaicking algorithm for a given tile (path-row). From the available pixels at the same coordinate, we retained those within 70% of the mean brightness, and selected the pixel exhibiting the median Normalized Difference Vegetation Index (NDVI). The corresponding images were processed through the CLASlite spectral mixture model, which yielded fractional cover of photosynthetic (PV), non-photosynthetic vegetation (NPV), and bare substrate within each image pixel. No data were available for some of the wettest regions in Panama (mainly ridge tops in the western part of the country), so we backfilled these areas with CLASlite results derived at course resolution using NASA Moderate Resolution Imaging Spectroradiometer (MODIS) data. The MODIS backfilling covered a total of 388,194 ha, or 5.2% of the country. The MODIS data were compiled over the year 2011 using daily MODIS data combined from the NASA Terra and Aqua satellite sensors, with bidirectional reflectance distribution function (BRDF) corrections and atmospheric compensation [56]. Prior to backfilling, the MODIS data were calibrated against the Landsat data using a linear transformation of reflectance values based on co-occurring Landsat and MODIS pixels (Additional file 1: Table S3).

We mosaicked two 90-m resolution NASA Shuttle Radar Topography Mission (SRTM) tiles to use as primary elevation data. From this mosaic, we used topographic modeling (3x3 kernel) to generate estimated slope and aspect at both 90-m resolution and 1-km resolution, with the latter generated from pixel-averaged SRTM elevation at 1-km resolution. Subsequent correlation analyses revealed that while higher resolution slope data were more predictive for LiDAR-observed carbon estimates than lower resolution slope data, 1-km aspect was more predictive than higher resolution aspect.

We used the most recent mean annual precipitation (mm yr^-1^) and seasonality (number of months yr^-1^ with rainfall below 100 mm) data from the NASA Tropical Rainfall Measuring Mission (TRMM) as of March 1, 2012 as additional inputs to the models for upscaling LiDAR data to the national level. Compared to Landsat and SRTM data, TRMM data are coarse (0.4° resolution) with a low signal to noise ratio. Thus, we generated smooth precipitation and seasonality estimates by applying a 5x5 median filter to each product. For RandomForest modeling (see below), we used the original climate data for calibrating the carbon model, and the smoothed datasets for mapped outputs. This option was not available for stratification, and thus we used the original climate data for both model calibration and mapping.

### National mapping

The LiDAR data collection effort was partitioned into three segments: (1) systematically aligned transects at the national scale; (2) additional flight lines as time and weather conditions allowed; and (3) large contiguous blocks for calibration and validation activities. All flights were conducted between 3 January 2012 and 1 February 2012 using CAO AToMS.

Twenty parallel LiDAR flight lines spaced 30 km apart were collected running on precise 0^o^ true North azimuths (Figure 1a). Based on the width of the country of Panama at each flight line, our transects ranged from approximately 60 to 200 km in length. The swath of each flight line, based on LiDAR settings described earlier, was 1.5 km. After cloud masking, the total systematically aligned data set covered 177,514 hectares, or 2.4% of the Panama’s land surface. As time and weather permitted, we added flight lines in areas deemed otherwise difficult to map due to persistent winds, clouds, and other factors (Figure 1a). These data covered a total area of 127,992 ha (an additional 1.7% of Panama).

We selected three large mapping areas containing field plots for use in either the LiDAR-to-ACD calibration or for purposes of regional mapping validation (Figure 1a). The total area of these polygons was 86,351 ha, and covered ecosystems ranging from montane wet forest, to lowland moist forest, to mesic and dry woodlands, shrublands and grasslands.

We stacked all LiDAR data and satellite input variables using ENVI image analysis software (Additional file 2: Figure S3). LiDAR, CLASlite, and SRTM datasets were aggregated to 1-ha resolution using pixel averaging, while TRMM data were resampled to 1-ha resolution with nearest neighbor selection. Thus, the 1-ha resolution data represent complete unmasked pixels for all data inputs in their native resolutions. Using LiDAR-derived carbon stocks as the dependent variable, we used two techniques to extend our results to the national level: stratification [11] and RandomForest machine learning [13]. Additional file 2: Figure S4 highlights interrelationships among the various input variables and LiDAR-derived ACD.

Stratification involves the subdivision of a region into unique classes, or strata, and the assignment of median, LiDAR-derived carbon estimates to those classes (Additional file 1: Table S4). The stratification was based on the SRTM, TRMM and CLASlite data, which produced 2039 classes. Of these, a clear majority of classes by total area (99.7% of Panama) were sampled with at least 1% LiDAR coverage (Additional file 3: Dataset S1). The remaining classes (0.3% of Panama) were reduced to simpler groupings based solely on PV –the variable most strongly related to carbon stocks in the preliminary analysis. Median carbon stock estimates for each class (as derived from LiDAR data) were then applied to all pixels within that class on nation-wide map.

RandomForest v4.7 (http://cran.r-project.org/web/packages/randomForest/index.html) is a non-parametric machine learning algorithm that produces a “forest” of decision trees based on random inputs from the training data [57]. In this case, we ingested an ordered sample of 29,508 ha, representing every 10th pixel of 1-ha resolution LiDAR coverage (Additional file 2: Figure S5). The ensemble tree was then selected upon which to base future predictions (Additional file 2: Figure S6, Additional file 4: Dataset S2). With continuous data, such as in this case, a regression-type tree was constructed. Because our LiDAR sampling was systematically planned, providing comprehensive spatial coverage across Panama, we allowed distance parameters (i.e., pixel position) to enter into the RandomForest algorithm in addition to our eight satellite variables. This had the effect of reducing large-scale spatial autocorrelation of modeling errors, although not eliminating them. This option was not tractable for the stratification approach because discretizing position variables (and intersecting them with environmental variables, as discussed above) resulted in an unreasonable number of unique classes.

To visualize how RandomForest ingests data and produces a predicted carbon map, we simulated the ingestion of a smaller amount of data (every 60th pixel) repeatedly to produce an animation (Additional file 5: Video S1). In the 24-fps video, each frame represents the accumulation of an additional 3 to 6 ha (depending on transect width) of LiDAR-based carbon estimates by the Carnegie Airborne Observatory (CAO). With each new data segment added to a cumulative dataset, RandomForest was re-run for each frame, producing a nation-wide simulated carbon map. The legend of the map corresponds to that in Figure 3; the inset shows the actual position of the plane during the flight campaign and the red dots show the magnitude of the LiDAR-estimated carbon stock at the plane’s position. As the CAO accumulates LiDAR data, the RandomForest model stabilizes on a final solution that incorporates all available LiDAR observations. The simulation is intended for visualization purposes only.

### Error

Error estimation in LiDAR-assisted mapping of ecosystem carbon is evolving rapidly, although several issues remain unresolved. The categories of errors are generally known; within the framework of LiDAR-assisted carbon mapping, sources of error include: (1) measurement of tree properties in the field (e.g., diameters, heights); (2) field-plot level predictions of carbon stocks from tree measurements and allometric models, including failure of allometric models to estimate real carbon stocks, and including plot size-edge effects [4]; (3) measurements by LiDAR remote sensing (e.g., integrated canopy height), including spatial and temporal plot co-location errors; (4) LiDAR-scale errors in predicted carbon stocks outside of field plots; (5) national- (or regional-) scale measurements of forest cover, elevation, etc.; and (6) prediction errors outside of LiDAR data.

These errors can be broken down into three basic sources, each depending on the accuracy and precision of a set of measurements and associated model predictions at the field-scale, LiDAR-scale, and national-scale. All of the above errors also depend on the spatial resolution of analysis, which varies widely across input variables used to achieve carbon mapping.

We follow previous LiDAR mapping studies at the global scale in excluding errors of category 1 and 2 (see above) in our map of error estimates [12,13]. Measurement errors of trees in the field are generally thought to make a vanishingly small contribution at the plot level [31,58], while allometric model errors in a single region are thought to be in the range of 10 to 30% of plot-scale carbon stock and can conceivably exceed this [25]. Moving forward, real carbon stock (i.e., that attained through direct harvest and weighing) will become the standard against which to assess the performance of either field-based or remote-sensing based carbon estimates [4]. At present, however, allometric errors applied to field data currently represent a “known unknown”, and thus field-estimated carbon stock (i.e., by allometric models) is currently considered to be the standard unit against which to compare subsequent model estimates [17,59]. This is particularly the case given the near universal reliance on a single family of tropical forest allometric equations (i.e., Chave et al. 2005 model 1). As remote-sensing assisted carbon mapping becomes the basic tool with which to assess carbon stocks, this lingering uncertainty will be addressed on a regional or ecosystem level with direct harvests and direct measurements of carbon stocks. At present, however, these errors will in principle affect field- and remote sensing-based carbon accounting in concert. Thus, any departures of Panamanian ecosystem carbon stock at the plot level from that predicted by the generalized Chave et al. (2005) framework used in this study would not be detectable even if every tree in Panama was measured using traditional field inventory.

For the remaining sources of error, we assessed “observed” errors using a conservative approach of setting aside validation data that was never used for LiDAR calibration or carbon mapping purposes (i.e., as opposed to iterative or leave-one out techniques). In our assessment of error, we do not rely at any point on the results of calibration model fits (e.g., RandomForest self-reported variance explained). Instead, we rely entirely on completely separate data left out of the original analysis. We use this data to determine how well our mapping effort performed. We considered observed errors in two steps: we considered what RMSE (predicted v. observed) was produced by the application of our LiDAR-to-carbon model in validation field plots, and what RMSE (predicted v. observed) was produced by our regional decision-tree model in the LiDAR samples used only for validation.

Errors in LiDAR measurement and prediction: We calibrated LiDAR using 57.7 ha of field plot data, setting aside 48.4 ha for validation purposes. Using validation plots (Figure 2b), we estimated errors of 10% (RMSE) at 1-ha plot size, consistent with previous results from other LiDAR studies at 1-ha resolution (Mascaro et al. 2011b, Zolkos et al. 2013).

Errors in satellite- (national-scale) measurement and prediction: We calibrated our national-scale modeling using 305,506 ha of transect and other flightline LiDAR data, setting aside 86,351 ha of LiDAR data with which to validate national-scale aboveground carbon modeling, both in a subset of particular ecoregions of interest (Figure 5, Additional file 1: Table S5), and within the entire validation area. Within the entire validation area, 62% of carbon stock variation was explained by RandomForest modeling (Additional file 2: Figure S7 and S8). Quantile regression indicated that RandomForest errors increased with increasing carbon stock (i.e., errors were heteroskedastic), both in terms of variance (red lines in Additional file 2: Figure S7) and a slight amount of bias (short black lines compared to the 1:1 line in Additional file 2: Figure S7). We fit a 3rd-order polynomial to the RMSE pattern to characterize these errors (Additional file 2: Figure S9), and applied this model to the predicted carbon stock map to estimate regional mapping errors.

Mapping of error estimates: For areas directly mapped by LiDAR, we applied only LiDAR-to-carbon errors at the pixel level (i.e., the direct LiDAR estimate of carbon being known with greater confidence for these pixels). For areas outside of direct LiDAR mapping, we applied both LiDAR-to-carbon and national-scale errors to the remaining pixels, by propagating the square root of the sum of each squared error value for each pixel according to:

$$\begin{array}{c} \epsilon_{\mathrm{National}\left(\mathrm{LiDAR}\;\mathrm{pixel}\right)}=\epsilon_{\mathrm{LiDAR}}\\ \epsilon_{\mathrm{National}\left(\mathrm{other}\;\mathrm{pixel}\right)}=\sqrt{\epsilon_{\mathrm{LiDAR}}^{2}+\epsilon_{\mathrm{DecisionTree}}^{2}} \end{array}$$

In doing so, we assumed that LiDAR calibration and regional mapping errors were independent.

Our error estimates, while informed by extensive validation data both in the form of field plots and LiDAR validation samples, should be viewed as initial estimates, rather than real measures of uncertainty. Refinements in uncertainty will come from improving our understanding of allometric equations used to estimate biomass [4] and chemical analyses of biomass carbon content [33]. Our analysis may also be improved through analytical modeling of errors (i.e., model-based inference) produced by both the LiDAR-to-carbon and national-scale models [60], as well as an improved understanding of spatial autocorrelation of errors. We explored simultaneous autoregressive modeling as a third upscaling technique (e.g., [41]), but at present the approach remains too computationally intensive to operate over an area of this size.

## Abbreviations

ACD: Aboveground carbon density; AToMS: Airborne Taxonomic Mapping System; AS: Agua Salud; AZ: Azuero; BCI: Barro Colorado Island; CAO: Carnegie Airborne Observatory; COL: Colon; CLASlite: Carnegie Landsat Analysis System-light; SHRM: Fort Sherman; LiDAR: Light Detection and Ranging; MCH: Mean canopy profile height; MODIS: Moderate Resolution Imaging Spectroradiometer; NASA: National Aeronautics and Space Administration; NPV: Non-photosynthetic vegetation; PV: Photosynthetic vegetation; REDD+: Reduced Emissions from Deforestation and Forest Degradation-plus; RMSE: Root mean squared error; SRTM: Shuttle Radar Topography Mission; TCH: Top-of-canopy height; TRMM: Tropical Rainfall Measuring Mission.

## Competing interests

The authors declare that they have no competing interests.

## Authors’ contributions

GA and JM designed the study. GA, JM, CA, DK, RM, and TK carried out airborne and satellite data collection and/or modeling. MVB, SD, JH, HML, CP, WS, JW and EB provided field data. GA and JM wrote the paper, and all other authors provided comments on the paper. All authors read and approved the final manuscript.

## Supplementary Material

Additional file 1: Tables S1-S5 This file provides tables on field plots, allometrics, national modeling, and validation. Click here for file

Additional file 2: Figures S1-S9 This file provides figures on field plot location, tree diameter-to-height relationships, satellite data inputs, national-scale modeling results, and validation results. Click here for file

Additional file 3: Dataset S1 Stratification data. Click here for file

Additional file 4: Dataset S2 RandomForest data. Click here for file

Additional file 5: Video S1 This file provides a video-based visualization of how the national-scale modeling ingests and responds to increasing amount of LiDAR-assisted carbon density estimation. Click here for file
