# Supplementary material for: High-fidelity national carbon mapping for resource management and REDD+
Source: Carbon Balance Manag. 2013 Jul 16;8:7. doi: 10.1186/1750-0680-8-7 (PMC3717137; doi:10.1186/1750-0680-8-7)
Supplement: Additional file 1: Tables S1-S5 — This file provides tables on field plots, allometrics, national modeling, and validation. [file 1750-0680-8-7-S1.pdf]

Additional File 1

Tables S1-S5. This file provides tables on field plots, allometrics, national modeling, and validation.

Table S1. Summary of field plots used to calibrate and validate LiDAR-based observations of forest structure. Plot configurations for BCI, SHRM, and GIG were 0.36 ha, 0.40-0.48 ha, and 0.80-1.00 ha respectively. We retained the configuration used by Mascaro et al. (2011a) for BCI that arbitrarily excludes a small portion of this 50 ha plot, which is not perfectly divisible by 0.36 ha. Mascaro et al. (Mascaro et al. 2011b) consider the variation in plot size and shape in detail. With greater understanding of the associated LiDAR calibration errors, Asner and Mascaro (submitted) utilized partitions of the SHRM an GIG plots at larger scales. Several 0.04 ha quadrats of the SHRM plot were excluded because their LiDAR height data were contaminated by the 60m+ canopy crane.

Area	Notes	Number of plots	Total area (ha)
Calibration			
Barro Colorado Island (BCI)	Old-growth and old secondary moist forests	128*	46.1
Agua Salud (AS)	Secondary moist forest, 1 to ~ 50 years of age	100	11.6
Validation			
Gigante (GIG)	Old-growth and old secondary moist forests	40*	38.4
Sherman (SHRM)	Old-growth and old secondary wet forests	13*	5.4
Azuero (AZ)	Secondary dry forest	33	3.3
Colon (COL)	Mangrove forest	5	1.3

*Single large mapped plot, apportioned into smaller sections.

Table S2. Summary of allometries used to estimate field-level aboveground carbon stocks, in each case following the Chave et al. (2005) "model 1" framework. AGB is aboveground biomass (kg) and H is height (m). Polynomial D to H model is based on unpublished data for Central Panama by J. Wright, fitting described in Asner et al. (2012). This model was also used for nearby wet forests at SHRM. For dry and mangrove forests, no field inventory height data was available; in these cases, maximum LIDAR height in each plot was fit as a power function of maximum diameter, and the resulting equation was used as a surrogate diameter-height allometry (fit using a non-arithmetic error term in the model, see Mascaro et al. 2011c) to prevent overestimation of AGB (Feldpausch et al. 2012).

**Ecotype**	**Model**	**Notes**
Wet forests (SHRM)		
	$AGB = 0.0776(\rho D^2 H)^{0.940}$	Chave et al. (2005)
	$H = \exp(0.862 + 0.611\ln(D) + 0.051\ln(D)^2 - 0.013\ln(D)^3)*1.0253$	Asner et al. (2012)
Moist forests (BCI, AS, GIG)		
	$AGB = 0.0509(\rho D^2 H)$	Chave et al. (2005)
	$H = \exp(0.862 + 0.611\ln(D) + 0.051\ln(D)^2 - 0.013\ln(D)^3)*1.0253$	Asner et al. (2012)
Dry forests (AZ)		
	$AGB = 0.112(\rho D^2 H)^{0.916}$	Chave et al. (2005)
	$H = 3.5409D^{0.3900}$	This study, LiDAR assisted
Mangrove forests (COL)		
	$AGB = 0.0509(\rho D^2 H)$	Chave et al. (2005)
	$H = 2.8407D^{0.5464}$	This study, LiDAR assisted

Table S3. Linear models used to re-scale MODIS CLASlite results, based on an subset of 107,650 co-occurring MODIS and Landsat pixels at 1-ha resolution collected adjacent to the greatest density of masked Landsat pixels (i.e., the areas nearest to areas without Landsat data in which MODIS data would be used). All models were significant at P < 0.0001. Values predicted to have negative fractional cover were forced to zero.

**MCU component**	**Model**	**Adj R2**
Soil	*Landsat* = 5.7096*MODIS* + 0.3318	0.07
Photosynthetic Veg (PV)	*Landsat* = -33.4624*MODIS* + 1.2497	0.54
Non-photosynthetic Veg (NPV)	*Landsat* = 10.9643*MODIS* + 0.9756	0.40

Table S4. Bins used to produce the primary stratification of environmental variables in Panama (2039 unique classes; see Appendix 1). Soil, PV, and NPV reflect fractional cover of soil, photosynthetic vegetation, and non-photosynthetic vegetation produced by CLASlite (see Fig. 1b). Elevation, slope and aspect are derived products from the Shuttle Radar Topography Mission (SRTM), while precipitation and seasonality are derived, annual products from the Tropical Rainfall Mapping Mission (TRMM; Fig. S3). In the later case, seasonality reflects the number of months per year estimated to have < 100 mm of precipitation. Brackets represent inclusive bins, while parentheses represent exclusive bins.

Soil	PV	NPV	Elevation	Slope	Aspect	Precipitation	Seasonality
[0 , 10)	[0 , 20)	[0 , 20)	[0 , 100)	[0 , 15)	[-1 , 0)	[0 , 2000)	[0 , 4)
[10 , inf)	[20 , 40)	[20 , inf)	[100 , 500)	[15 , 45)	[0 , inf)	[2000 , 4000)	[4 , inf)
	[40 , 60)		[500 , 1500)	[45 , inf)		[4000 , inf)	
	[60 , 80)		[1500 , inf)				
	[80 , 90)						
	[90 , 95)						
	[95 , 98)						
	[98 , 99)						
	[99 , inf)						

Table S5. Six arbitrarily selected target ecoregions used for validation of stratification and decision tree modeling of carbon stocks in Panama. Bias and noise for these regions is shown in the main text (Fig. 5). Including these targeted ecoregions, a total of 86,351 ha of LiDAR data were used for validation (Fig. S6, S7).

Region	Area (ha)
Azuero (dry secondary forests)	6,711
Agua Salud (young secondary moist forests)	3,264
Fortuna (montane wet forests)	6,680
Colon (moist mangrove forests)	1,012
Sherman (low elevation wet forests)	3,910
BCI (low elevation moist forests)	1,059
