# Supplementary material for: High-fidelity national carbon mapping for resource management and REDD+
Source: Carbon Balance Manag. 2013 Jul 16;8:7. doi: 10.1186/1750-0680-8-7 (PMC3717137; doi:10.1186/1750-0680-8-7)
Supplement: Additional file 2: Figures S1-S9 — This file provides figures on field plot location, tree diameter-to-height relationships, satellite data inputs, national-scale modeling results, and validation results. [file 1750-0680-8-7-S2.pdf]

Additional file 2

Figures S1-S9. This file provides figures on field plot location, tree diameter-to-height relationships, satellite data inputs, national-scale modeling results, and validation results.

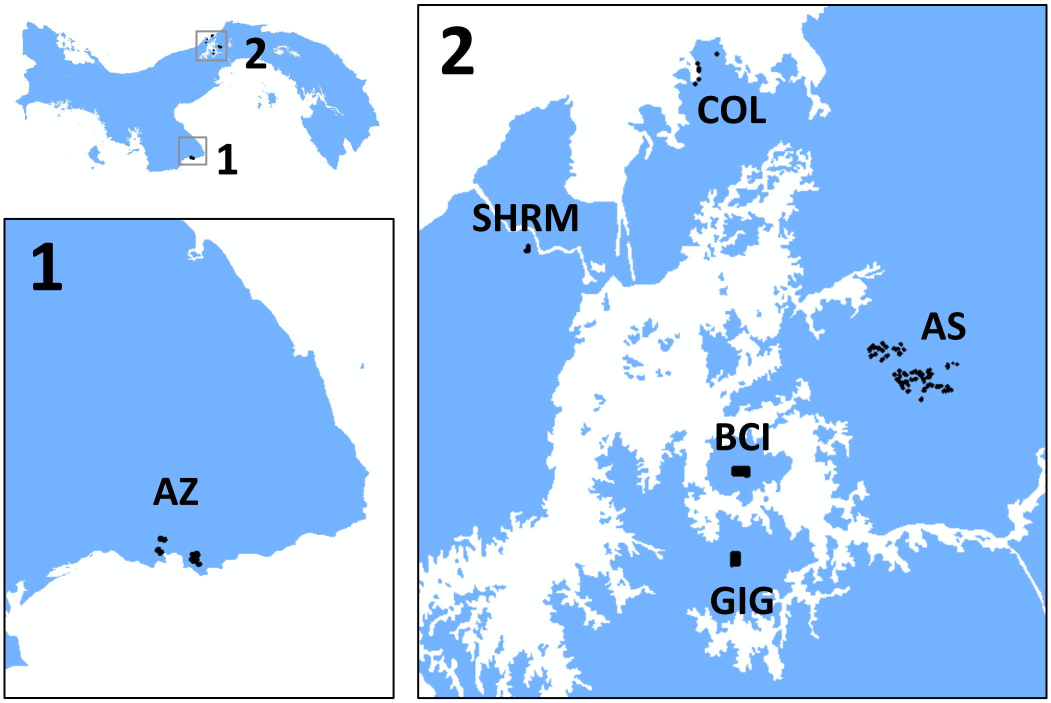

Figure S1. Sources of field data: old-growth moist forests on Barro Colorado Island (BCI) and secondary moist forests within Agua Salud (AS) were used to calibrate the LiDAR-field carbon model. We subsequently compared the predictions of this model to additional field-based estimates of carbon in mixed old-growth and old secondary forests at Fort Sherman (SHRM), dry secondary forests on the Azuero Peninsula (AZ), and mangrove forests in Colon (COL).

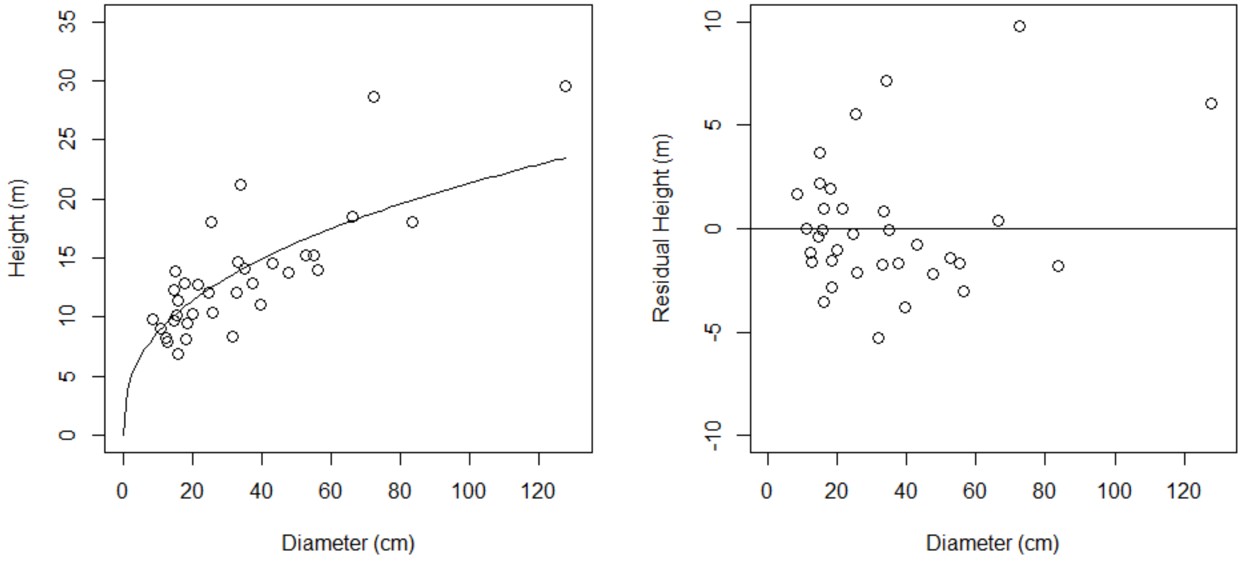

Figure S2. Height-diameter constraint for dry forests in Azuero. In this case, given the lack of height information from field inventory data, maximum LiDAR top-of-canopy height for each transect was compared to maximum tree diameter. This "constraint" of height estimates was essential to prevent overestimation that is ubiquitous in the absence of height data or a height-diameter model.

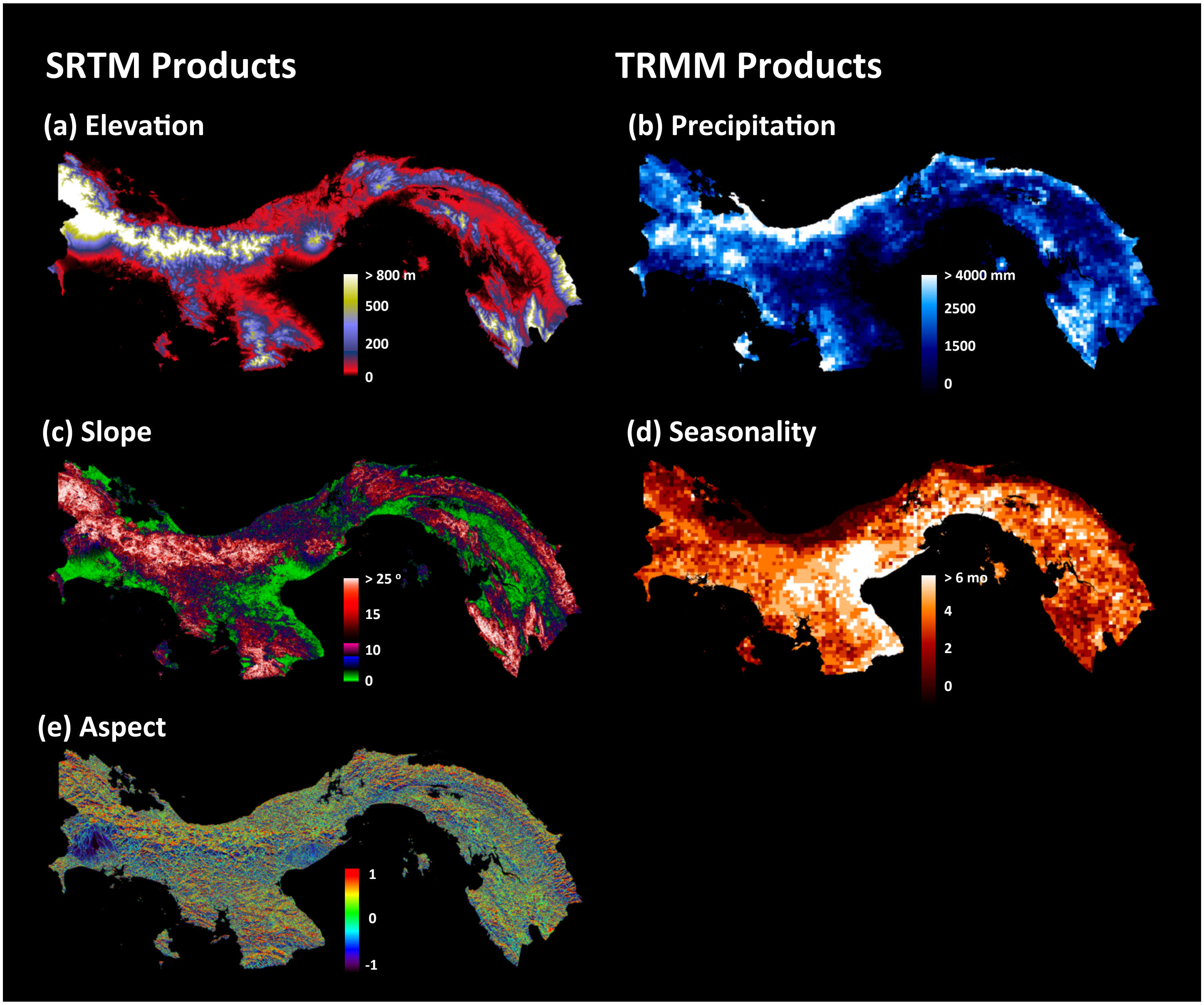

Figure S3. Elevation (m a.s.l.), slope (degrees) and aspect (cosine-transformed bearing) are derived products from the Shuttle Radar Topography Mission (SRTM), while precipitation (mm per year) and seasonality (number of months per year estimated to have < 100 mm of precipitation) are derived, annual products from the Tropical Rainfall Mapping Mission (TRMM).

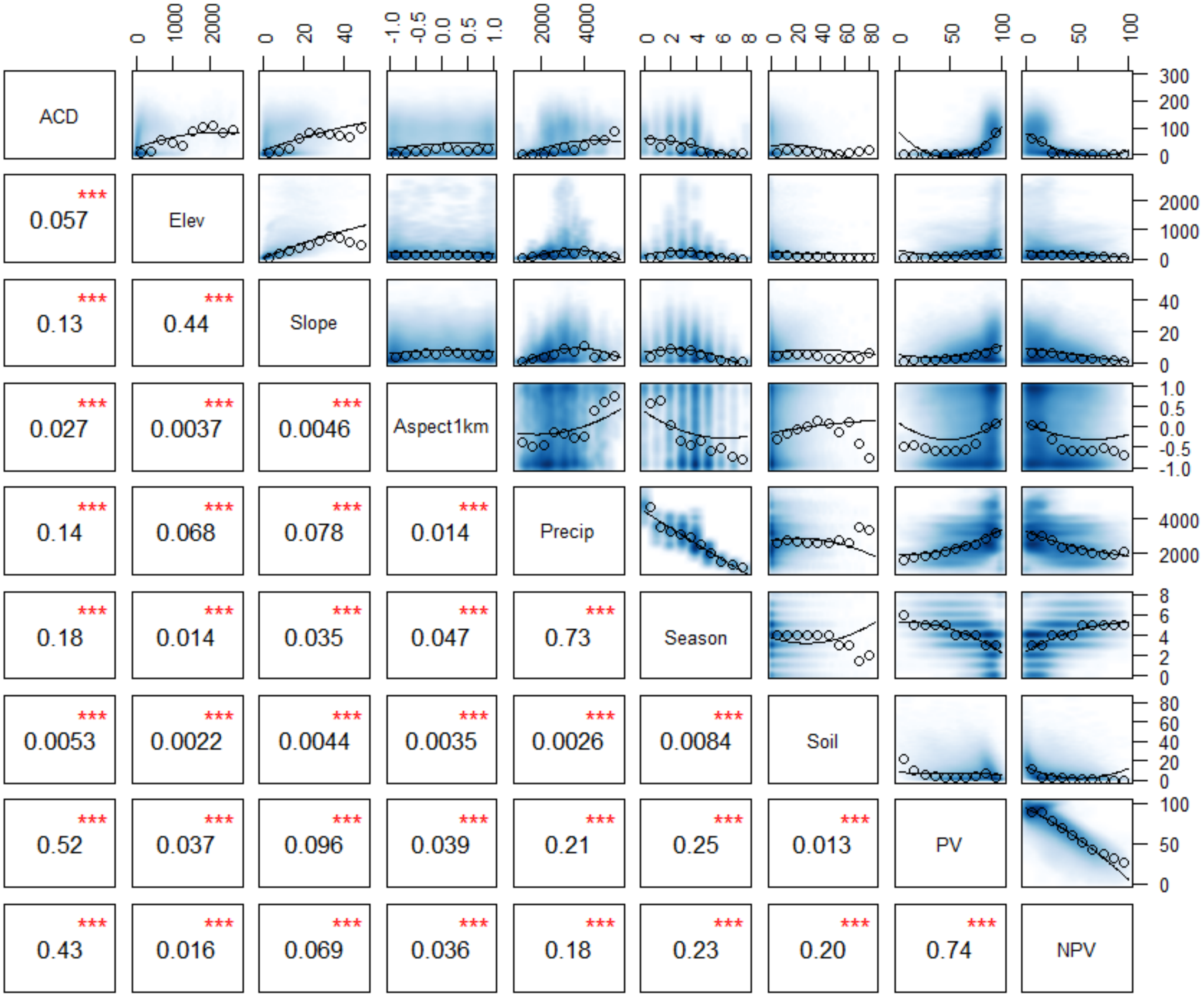

Figure S4. A scatterplot matrix demonstrating variation among 1-ha resolution LiDAR-based aboveground carbon density (ACD) estimates and eight geospatially explicit satellite variables. Soil, PV and NPV are percentage fractional cover, derived products from CLASlite; elevation (m), slope (degrees) and aspect (cosine-transformed bearing) are derived products from the Shuttle Radar Topography Mission (SRTM); and precipitation (mm yr^{-1})and seasonality are derived, annual products from the Tropical Rainfall Mapping Mission (TRMM). In the later case, seasonality reflects the number of months per year estimated to have < 100 mm of precipitation. The blue scatter reflects point densities of the 305,506 ha of LiDAR calibration data. The black circles are binned medians, and the solid lines are second-order polynomial models. Statistics are Pearson's R. The scatterplots, binned medians, and polynomial models are shown only for reference and did not enter into national scale modeling in any way.

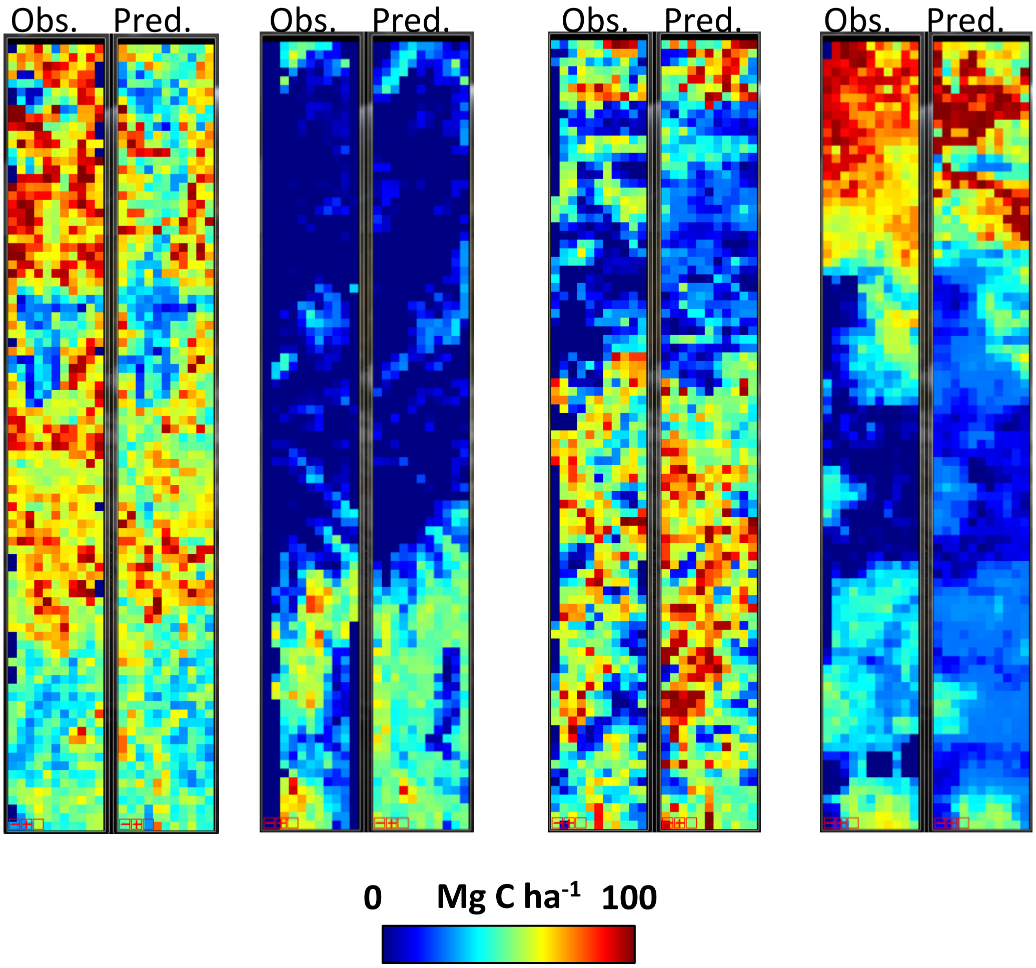

Figure S5. Predicted (random forest) versus LiDAR-observed aboveground carbon density in example LiDAR transects.

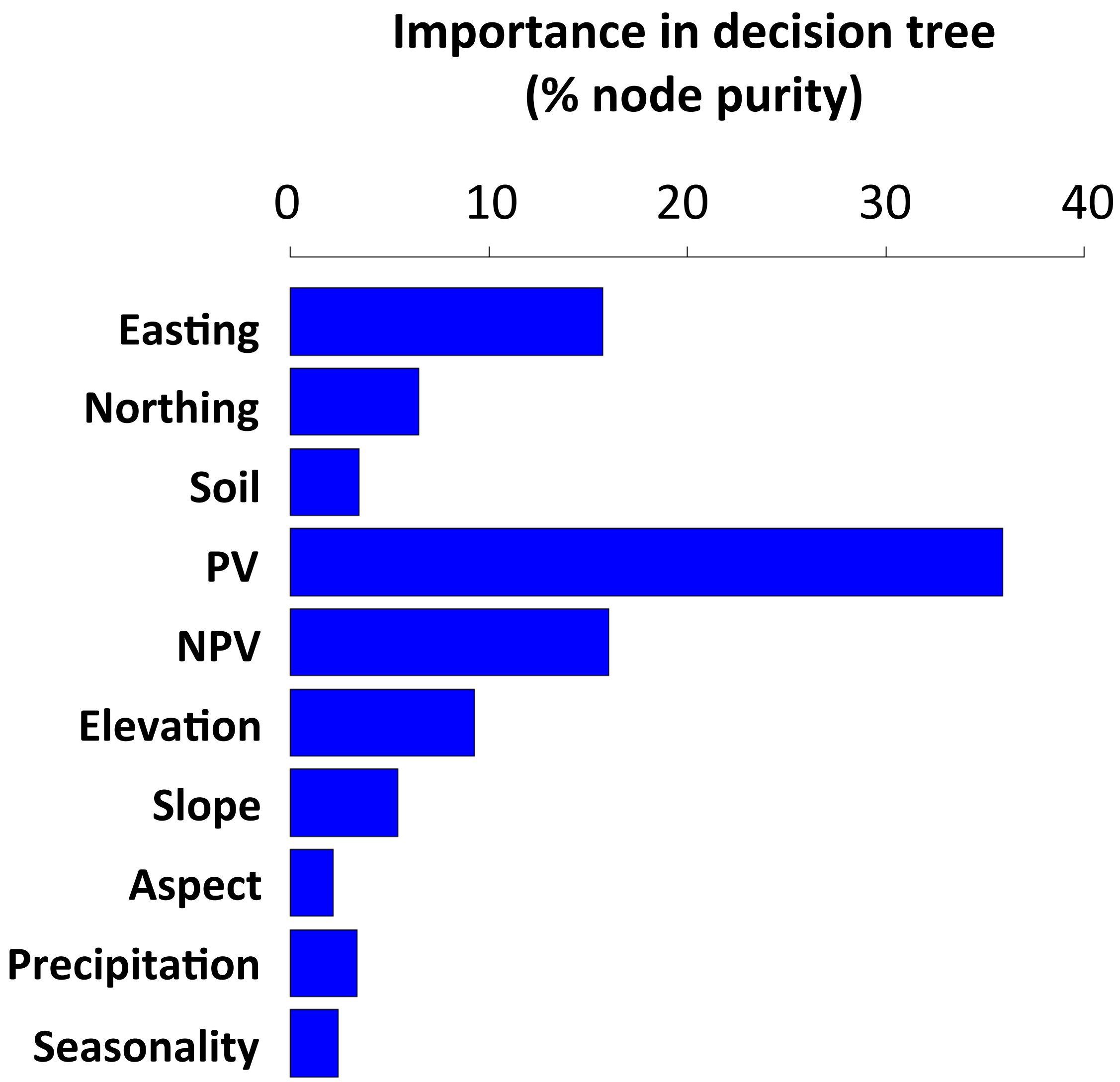

Figure S6. The relative importance of ten input variables in the ensemble tree produced by the RandomForest machine learning algorithm.

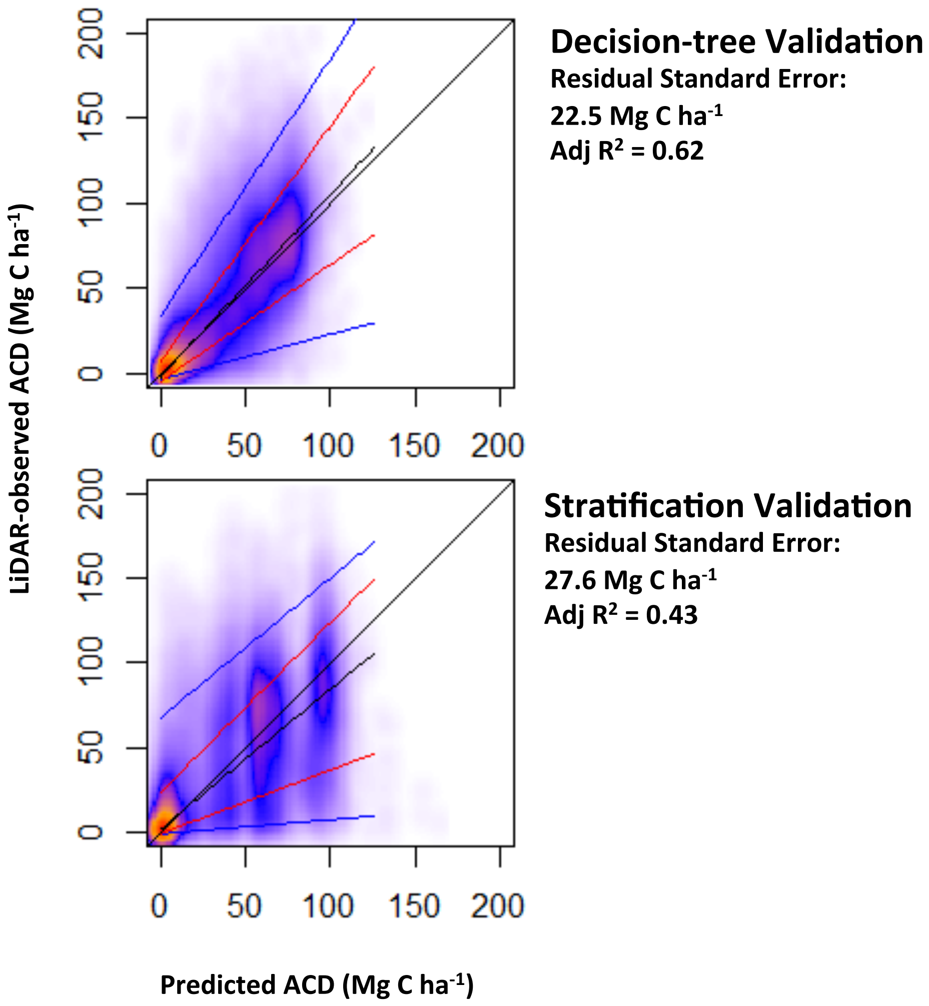

Figure S7. Predicted versus observed ACD in three large validation areas totaling 86,334 ha flown with CAO LiDAR and set aside solely for validation. These areas represent six distinct ecoregions (see main text). The scatterplot highlights increasing point density from blue to yellow to red. Short lines represent quantiles of tau = 0.975 (blue, upper), 0.84 (red, upper), 0.5 (black), 0.16 (red, lower), and 0.025 (blue, lower) ; where these tau values are the percentiles of LiDAR-observed values about the model predictions. The longer black line is 1:1.

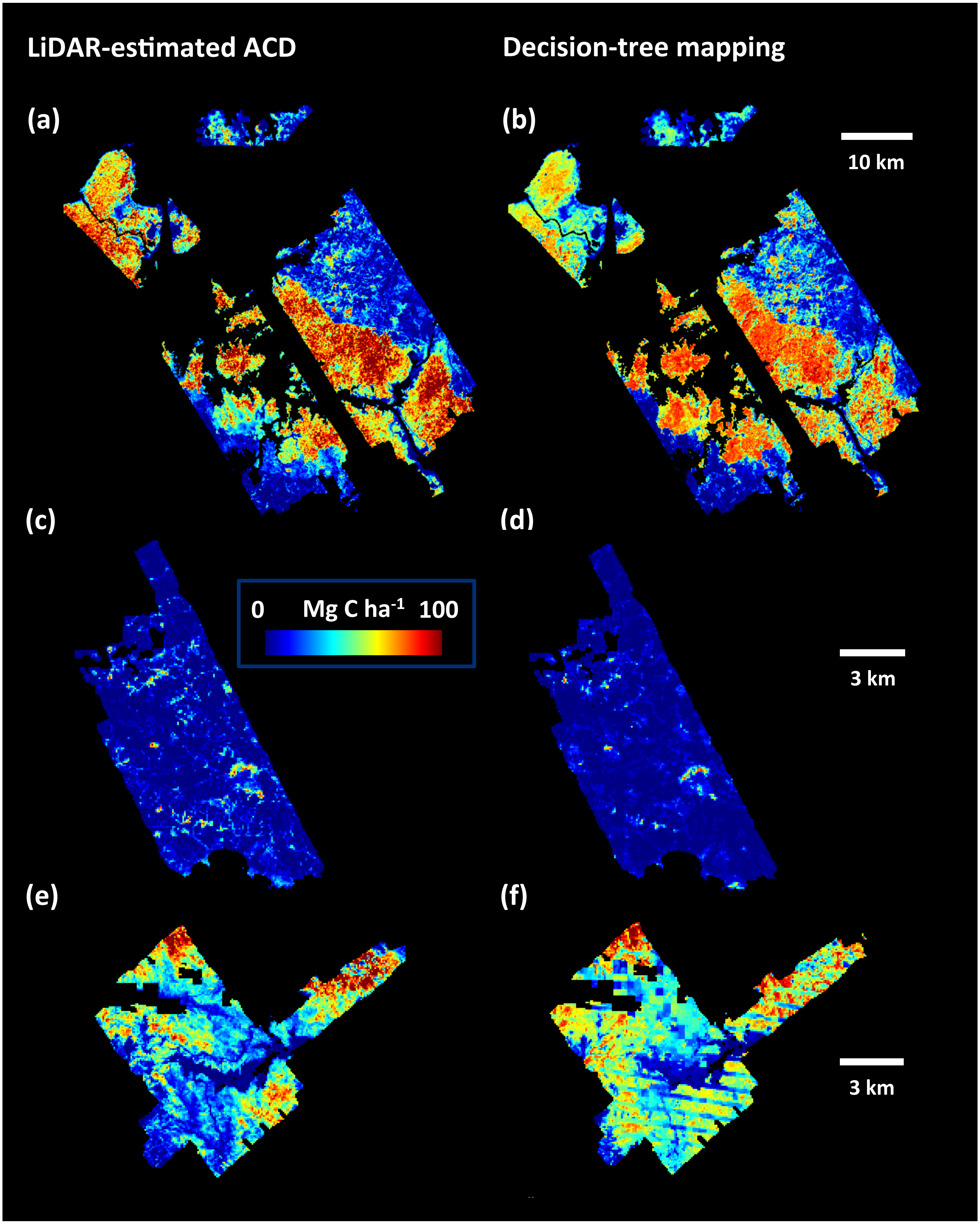

Figure S8. Airborne LiDAR validation of regional mapping. LiDAR-estimated carbon densities (left) are compared to estimates from decision-tree mapping (right) for the Panama canal area (a, b), Azuero dry forests (c, d), and Fortuna high-elevation wet forests (e, f).

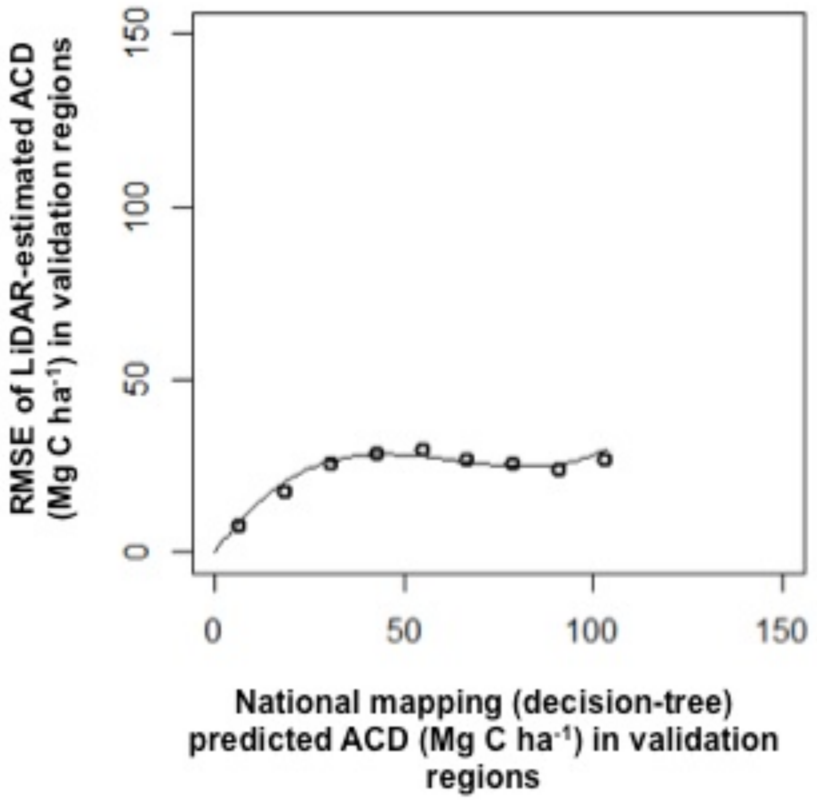

Figure S9. Spatially explicit uncertainty estimates for national (decision-tree) mapped aboveground carbon density (ACD). Circles represent binned means at 10 Mg intervals, while the curve is a 3rd-order polynomial forced through the origin.
