# Supplementary material for: High-fidelity national carbon mapping for resource management and REDD+
Source: Carbon Balance Manag. 2013 Jul 16;8:7. doi: 10.1186/1750-0680-8-7 (PMC3717137; doi:10.1186/1750-0680-8-7)
Supplement: Additional file 5: Video S1 — This file provides a video-based visualization of how the national-scale modeling ingests and responds to increasing amount of LiDAR-assisted carbon density estimation. [file 1750-0680-8-7-S5.pptx]

## Slide 1
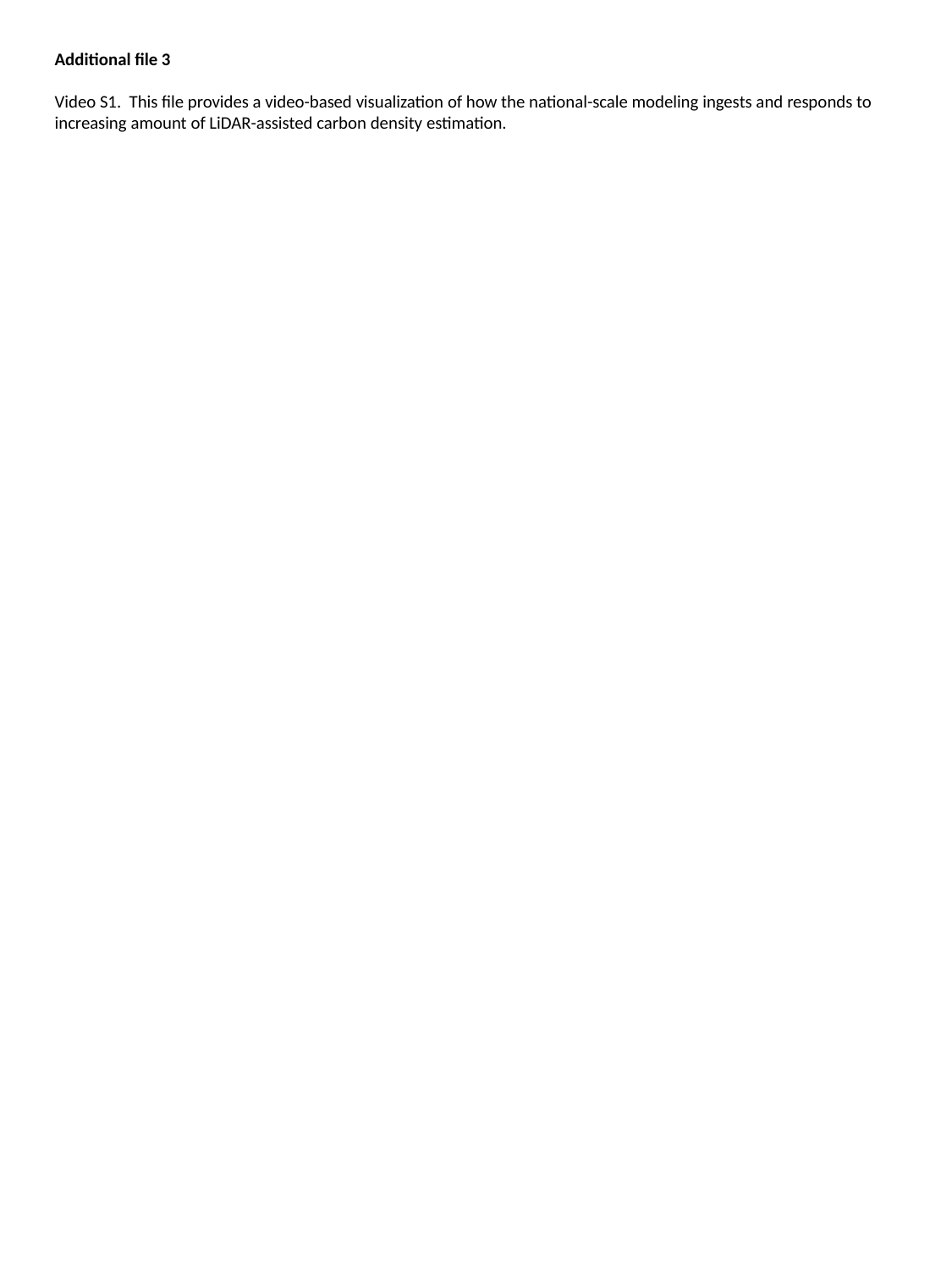

Additional file 3
Video S1. This file provides a video-based visualization of how the national-scale modeling ingests and responds to increasing amount of LiDAR-assisted carbon density estimation.

## Slide 2
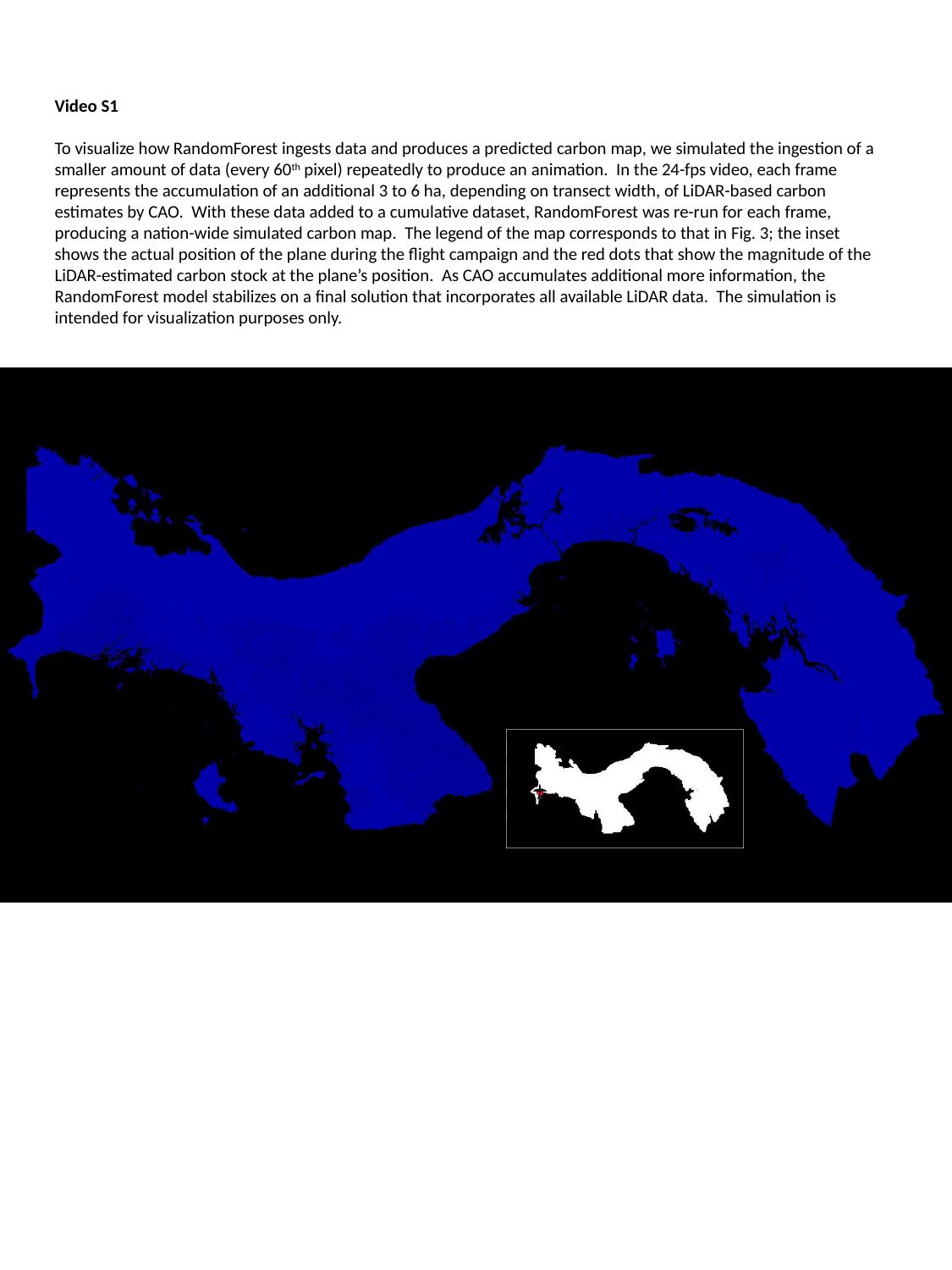

Video S1
To visualize how RandomForest ingests data and produces a predicted carbon map, we simulated the ingestion of a smaller amount of data (every 60th pixel) repeatedly to produce an animation. In the 24-fps video, each frame represents the accumulation of an additional 3 to 6 ha, depending on transect width, of LiDAR-based carbon estimates by CAO. With these data added to a cumulative dataset, RandomForest was re-run for each frame, producing a nation-wide simulated carbon map. The legend of the map corresponds to that in Fig. 3; the inset shows the actual position of the plane during the flight campaign and the red dots that show the magnitude of the LiDAR-estimated carbon stock at the plane’s position. As CAO accumulates additional more information, the RandomForest model stabilizes on a final solution that incorporates all available LiDAR data. The simulation is intended for visualization purposes only.
